# Negative membrane capacitance of outer hair cells: electromechanical coupling near resonance

**DOI:** 10.1038/s41598-017-12411-6

**Published:** 2017-09-21

**Authors:** Kuni H. Iwasa

**Affiliations:** 35A Convent Dr., Rm 1F242A, NIDCD, National Institutes of Health, Bethesda Maryland, 20892 USA

## Abstract

Outer hair cells in the cochlea have a unique motility in their cell body based on mechanoelectric coupling, with which voltage changes generated by stimuli at their hair bundles drive the cell body and, in turn, it has been assumed, amplifies the signal. *In vitro* experiments show that the movement of the charges of the motile element significantly increases the membrane capacitance, contributing to the attenuation of the driving voltage. That is indeed the case in the absence of mechanical load. Here it is predicted, however, that the movement of motile charges creates negative capacitance near the condition of mechanical resonance, such as those in the cochlea, enhancing energy output.

## Introduction

The exquisite sensitivity and the frequency bandwidth reaching as high as 100 kHz of mammalian hearing, depending on the animal species^1^, is based on the ability of its ear to function as a frequency analyzer^2^. The frequency components are then transmitted to the brain in parallel by a bundle of neurons. Thus a key question is how a system that is based on biological cells is capable of operating at such high frequencies.

For the mammalian ear to be a sensitive mechanoeletrical analyzer, it is essential to counteract viscous drag^3,4^ and outer hair cells (OHCs) play a key role^5,6^. These cells have a motile mechanism in their cell body based on piezoelectricity, called “somatic motility” or “electromotility”, which utilizes electrical energy^7–11^. The key component of this motile element is prestin, a member SLC26A5 of the SLC family of membrane proteins^12^. The electric potential that is used by the motile mechanism is generated by mechanotransducer current of the sensory hair bundles of these cells, responding to mechanical stimuli. This process is assisted by the endocochlear potential, the unusual positive potential in the K^+^-rich endolymphatic space, generated by the stria vascularis. Indeed, the electrical energy and the ionic environment provided to OHCs are exceptional. However, a question remains as to how OHCs can be effective at high frequencies: while viscous drag increases with the frequency, the receptor potential, which drives this motile mechanism, decreases with frequency by the capacitive conductance of the basolateral membrane^13^.

This puzzle has been called the “RC time constant” problem, the reason for a dispute regarding the basis for the amplifying role of OHCs: active process in the hair bundle alone^14^, or somatic motility coupled with hair bundle transduction^15,16^, or a combination of both^17^. The second point of view was examined by considering various mechanisms that could possibly improve the effectiveness of somatic motility^18–24^.

Despite their differences, all these previous analyses assume that the membrane capacitance, which consists of two components, linear and nonlinear, is unaffected by the mechanical load on OHCs. Of the two components, the linear component is structural, primarily based on the capacitance of the plasma membrane. The nonlinear component is due to the charge movement associated with the motile mechanism in the cell. This component has a bell-shaped membrane potential dependence under the load-free condition. Its peak value can be larger than the linear capacitance^7,8^. For this reason, the motor charge appears to enhance “RC attenuation” even further.

A recent analysis, however, showed that mechanical load, particularly viscous drag, decreases nonlinear capacitance and increases mechanical energy output of OHCs^25^. Here it is shown, using a simple model system, that the effect of mechanical resonance is even more substantial. It can fully nullify the membrane capacitance and increase the energy output of OHCs. The implications of this finding to the cochlea are discussed. The resulting inequality describes an upper bound of the effectiveness of OHCs.

## The Model System

**Figure 1:**
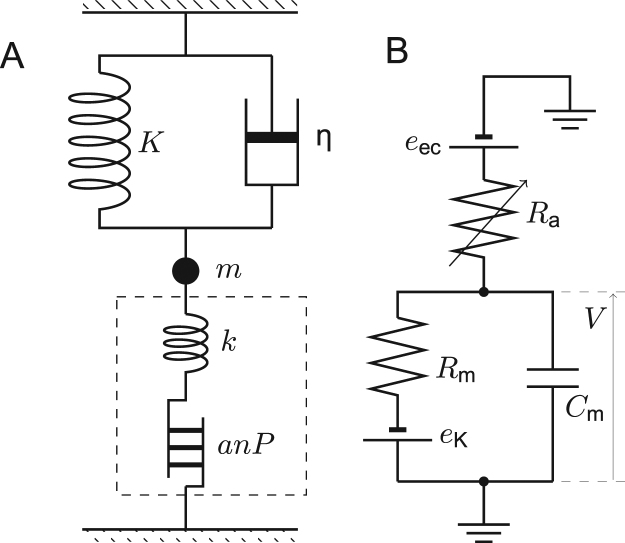
Mechanical connectivity (**A**) and the equivalent electric circuit of the model system (**B**). Changes in hair bundle conductance *R*_*a*_ drives the system. (**A)** intrinsic cell stiffness *k*, external elastic load *K*, mass *m*, drag coefficient *η*. The motile element changes the cell length by *x* = *k*/(*k* + *K*)⋅*anP*, where *P* represents the fraction of the motile elements in the elongated state. The quantities *a*, *q*, and *n* respectively represent unitary length change, the unitary charge change, and the number of motile units. The broken line indicates the border of the OHC. The connectivity of the cell and the external load are parallel because the magnitudes of their displacements are equal. (**B)** the membrane potential *V*, the basolateral resistance *R*_*m*_, the total membrane capacitance of the basolateral membrane *C*_*m*_. The endocochlear potential *e*_*ec*_, and the potential due to K^+^ permeability of the basolateral membrane *e*_*K*_. The apical capacitance is ignored.

Consider a simple model system, where an OHC is connected to a spring with stiffness *K*, a dashpot with friction coefficient *η*, and a mass *m* (Fig. 1). We assume here that the cell has *n* motile elements, which has two discrete states, compact and extended, and during a transition from the compact state to the extended state, the cell length increases by *a* and the electric charge *q* flips across the plasma membrane. The axial stiffness of the cell is *k*. The definitions of the parameters and the variables are given in Table 1 and in the caption to Fig. 1. The set of the equations for this system has been derived previously^25^.

**Table 1:** List of variables and parameters. For the notations of other parameters, please see Fig. 1.

notation	definition	unit
*α* ^2^	$1+\gamma {a}^{2}n\tilde{K}$	dimensionless
*β*	1/*k*_*B*_ *T*	1/J
*γ*	$\beta \overline{P}\mathrm{(1}-\overline{P})$	1/J
*ζ*	*γnq* ^2^/*C*_0_	dimensionless
*σ*	1/${\overline{R}}_{a}+\mathrm{1/}{R}_{m}$	S
*ω*_*r*_	$\sqrt{(k+K)/m}$	1/s
*ω*_*η*_	(*k* + *K*)/*η*	1/s
$\overline{\omega }$	*ω*/*ω*_*r*_	dimensionless
${\overline{\omega }}_{\eta }$	*ω*_*η*_/*ω*_*r*_	dimensionless
*C*_0_	regular capacitance	F
*i*_0_	$({e}_{ec}-{e}_{K})/({\overline{R}}_{a}+{R}_{m})$	A
$\tilde{K}$	*kK*/(*k* + *K*)	N/m
$\hat{r}$	relative amplitude of *R*_*a*_	dimensionless

Let *P* be the fraction of the motile units in the extended state. Its equilibrium value *P*_∞_ follows the Boltzmann distribution *P*_∞_ = 1/(1 + exp[*β*Δ*G*]), with *β* = 1/(*k*_*B*_ *T*), where *k*_*B*_ is Boltzmann’s constant and *T* the temperature, and ${\rm{\Delta }}G=q(V-{V}_{\mathrm{1/2}})+\tilde{K}{a}^{2}n(P-{P}_{0})$. Here $\tilde{K}=kK/(k+K)$; *V*_1/2_ and *P*_0_ are constants. If the system is not in equilibrium, *P*_∞_ is regarded as the target value, toward which *P* changes. Because cell displacement can be expressed by *k*/(*k* + *K*)⋅*anP* ^25^, the equation of motion turns into

1 $$m\frac{{d}^{2}P}{d{t}^{2}}+\eta \frac{dP}{dt}=(k+K)({P}_{\infty }-P),$$

for small difference between *P*_∞_ and *P*. In a special case of *m* = 0, Eq. 1 turns into a relaxation equation. The receptor potential *V* is determined by

2 $$\frac{{e}_{ec}-V}{{R}_{a}}=\frac{V-{e}_{K}}{{R}_{m}}+{C}_{0}\frac{dV}{dt}-nq\frac{dP}{dt}.$$

Here *R*_*a*_ is the apical membrane resistance, which is dominated by mechanotransducer channels in the hair bundle. The basolateral membrane has the resistance *R*_*m*_ and the linear capacitance *C*_0_, which is determined by the membrane area.

### Response to Small Oscillatory Stimuli

Let us assume that the hair bundle is stimulated with sinusoidal waveform with an angular frequency *ω*. The apical resistance responds at the same frequency ${R}_{a}(t)={\overline{R}}_{a}\mathrm{(1}+\hat{r}\exp [i\omega t\mathrm{])}.$ Other variables of the system respond by small periodic changes from their steady state values: $V(t)=\overline{V}+v\exp [i\omega t],\,{P}_{\infty }(t)=\overline{{P}_{\infty }}+{p}_{\infty }\exp [i\omega t],\,and\,P(t)=\overline{P}+p\exp [i\omega t\mathrm{]}.$ Here the variables in lower case letters are small and those marked with bars on top are time-independent. Hence $\bar{V}=({e}_{ec}{R}_{m}+{e}_{K}{\bar{R}}_{a})/({R}_{m}+{\bar{R}}_{a})$ and $\overline{P}=\overline{{P}_{\infty }}$.

The equations for the small amplitudes are given as

3a $${p}_{\infty }=-\gamma (qv+{a}^{2}n\tilde{K}p),$$

3b $$[-{(\omega /{\omega }_{r})}^{2}+i\omega /{\omega }_{\eta }]p=({p}_{\infty }-p),$$

3c $$-\frac{{e}_{ec}-\overline{V}}{{\overline{R}}_{a}}\hat{r}=(\frac{1}{{\overline{R}}_{a}}+\frac{1}{{R}_{m}})v+i\omega ({C}_{0}-nq\cdot p)v,$$

by introducing the resonance frequency ${\omega }_{r}(=\sqrt{(k+K)/m})$, the viscoelastic roll-off frequency *ω*_*η*_(=(*k* + *K*)/*η*) and a parameter $\gamma =\beta \overline{P}\mathrm{(1}-\overline{P})$, which depends on the operating point of the motile element.

Eq. 3c can be transformed into

4 $$-{i}_{0}\hat{r}=(\sigma +i\omega {C}_{0})v-i\omega nqp,$$

by introducing the steady state current ${i}_{0}=({e}_{ec}-{e}_{K})/({\bar{R}}_{a}+{R}_{m})$, and the steady-state conductance $\sigma \,=\,\mathrm{1/}{\overline{R}}_{a}+\mathrm{1/}{R}_{m}$.

The combination of Eqs 3a and 3b leads to,

5 $$[-{(\omega /{\omega }_{r})}^{2}+i\omega /{\omega }_{\eta }+{\alpha }^{2}]p=-\gamma qv,$$

where ${\alpha }^{2}\mathrm{=1}+\gamma {a}^{2}n\tilde{K}$. For the list of these parameters, see Table 1.

**Figure 2:**
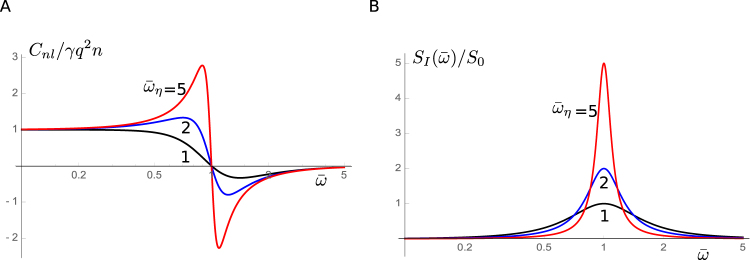
Nonlinear capacitance *C*_*nl*_ and power spectral density *S*_*I*_(*ω*) of current noise. (**A)** Nonlinear capacitance plotted against $\overline{\omega }(=\omega /{\omega }_{r})$. Nonlinear capacitance *C*_*nl*_ is normalized by *γnq* ^2^. (**B)** Power spectral density of current noise is plotted against $\overline{\omega }$. ${S}_{I}(\overline{\omega })$ is normalized by ${S}_{0}\mathrm{(=4}\overline{P}\mathrm{(1}-\overline{P})n{q}^{2}{\omega }_{r})$. Traces respectively correspond to the values of ${\overline{\omega }}_{\eta }$: 1 (black), 2 (blue), and 5 (red).

The contribution *C*_*nl*_ of the motor charge to the membrane capacitance *C*_*m*_ is given by *C*_*nl*_ = (*nq*/*v*)*Re*[*p*]. This leads to, *C*_*m*_ = *C*_0_ + *C*_*nl*_ with

6 $${C}_{nl}=\frac{\gamma n{q}^{2}[{\alpha }^{2}-{\overline{\omega }}^{2}]}{{[{\alpha }^{2}-{\overline{\omega }}^{2}]}^{2}+{(\overline{\omega }/{\overline{\omega }}_{\eta })}^{2}},$$

where $\overline{\omega }=\omega /{\omega }_{r}$, ${\overline{\omega }}_{\eta }={\omega }_{\eta }/{\omega }_{r}$, and *C*_0_ is the regular membrane capacitance, which is proportional to the membrane area of the cell (Fig. 2A). Eq. 6 leads to *C*_*nl*_ = *γnq* ^2^ in the absence of mechanical load, consistent with earlier studies^25–27^.

Nonlinear capacitance is associated with current noise. Voltage oscillation *v* exp[*iωt*] generates current *iωnqp* exp[*iωt*]. The admittance is given by *Y*(*ω*) = *iωnqp*/*v*. Since Johnson-Nyquist noise^28,29^ is related to the admittance with the formula *S*_*I*_(*ω*) = 4*k*_*B*_ *T Re*[*Y*(*ω*)], we have

7 $${S}_{I}(\omega )=\frac{4\overline{P}\mathrm{(1}-\overline{P})n{q}^{2}\cdot \overline{\omega }/{\overline{\omega }}_{\eta }\cdot \omega }{{[{\alpha }^{2}-{\overline{\omega }}^{2}]}^{2}+{(\overline{\omega }/{\overline{\omega }}_{\eta })}^{2}},$$

for the power spectral density. It has a peak $4\overline{P}\mathrm{(1}-\overline{P})n{q}^{2}{\omega }_{\eta }$ at $\overline{\omega }=\alpha$ (Fig. 2B). This spectral shape is quite different from that without mechanical resonance, which has high-pass characteristics^30,31^.

Let us examine the power output elicited by hair bundle stimulation. Since the voltage change *v* is the result of a change *r* in the hair bundle resistance as described by Eq. 4, it is expressed by

8 $$v=\frac{-{i}_{0}\hat{r}+i\omega nqp}{\sigma +i\omega {C}_{0}}.$$

By combining Eqs 5 and 8, we obtain

9 $$[-{(\frac{\omega }{{\omega }_{r}})}^{2}+i\omega (\frac{1}{{\omega }_{\eta }}+\frac{\gamma n{q}^{2}}{\sigma +i\omega {C}_{0}})+{\alpha }^{2}]p=\frac{\gamma q{i}_{0}}{\sigma +i\omega {C}_{0}}\hat{r}.$$

## Results

### Power Output at High Frequencies

**Figure 3:**
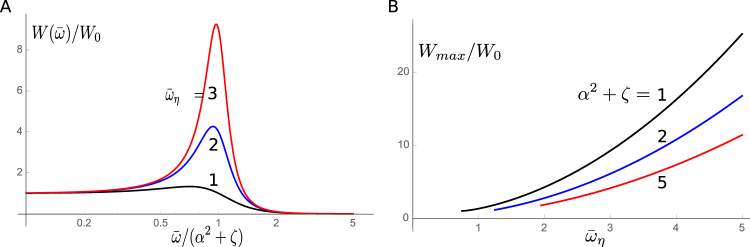
Power output per unit resistance change $(\hat{r}=\mathrm{1)}$. (**A**) Frequency dependence of power output. The reduced frequency $\overline{\omega }$ is normalized by *α* ^2^ + *ζ*. Power output $W(\overline{\omega })$ is normalized by ${W}_{0}=\gamma \zeta {a}^{2}n{i}_{0}^{2}\eta {k}^{2}\mathrm{/[2}\pi {(k+K)}^{2}{C}_{0}]$. Traces correspond to the values of ${\overline{\omega }}_{\eta }$: 1, (black); 2, (blue); and 3 (red). (**B**) Maximum power output plotted against ${\overline{\omega }}_{\eta }(={\omega }_{\eta }/{\omega }_{r})$. The scale of power output is the same as in A. Traces correspond to the values of *α* ^2^ + *ζ*: 1, (black); 1.5, (blue); and 2 (red).

Since we are interested is in high frequency range, we may assume *σ* + *iωC*_0_ → *iωC*_0_. Under this condition, the capacitance ratio, *ζ* = *γnq* ^2^/*C*_0_, becomes a useful parameter. The work against drag per half cycle is ${E}_{d}=\mathrm{(1/2)}\eta \omega {(\tilde{K}/K)}^{2}|nap{|}^{2}$. Power output *W*_*d*_ = 2*ω*/(2*π*)*E*_*d*_ (Fig. 3A) is maximized at ${\overline{\omega }}^{2}={\alpha }^{2}+\zeta -\mathrm{1/(2}{\overline{\omega }}_{\eta }^{2})$ and the maximal value is (Fig. 3B),

10 $${W}_{d}^{(max)}=\frac{4\gamma \zeta {a}^{2}n{i}_{0}^{2}{\overline{\omega }}_{\eta }^{4}}{\mathrm{4(}{\alpha }^{2}+\zeta ){\overline{\omega }}_{\eta }^{2}-1}\cdot \frac{\eta {k}^{2}{\hat{r}}^{2}}{2\pi {(k+K)}^{2}{C}_{0}},$$

using a reduced frequency $\overline{\omega }=\omega /{\omega }_{r}$, and ${\overline{\omega }}_{\eta }={\omega }_{\eta }/{\omega }_{r}$.

If ${\overline{\omega }}_{\eta }$ is sufficiently large to satisfy $\mathrm{4(}{\alpha }^{2}+\zeta ){\overline{\omega }}_{\eta }^{2}\gg 1$, it can be approximated by

11 $${W}_{d}^{(max)}\approx \frac{\gamma \zeta {a}^{2}n{i}_{0}^{2}{\overline{\omega }}_{\eta }^{2}}{{\alpha }^{2}+\zeta }\cdot \frac{\eta {k}^{2}{\hat{r}}^{2}}{2\pi {(k+K)}^{2}{C}_{0}}.$$

### Negative Capacitance

**Figure 4:**
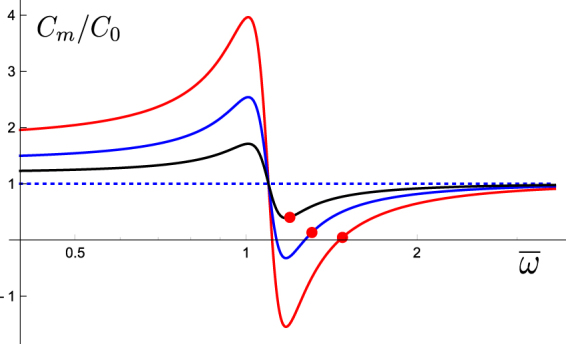
Membrane capacitance near resonance. The membrane capacitance *C*_*m*_(=*C*_0_ + *C*_*nl*_) normalized to the linear capacitance *C*_0_ is plotted against the normalized frequency $\overline{\omega }(=\omega /{\omega }_{r})$. Here the ratio *ζ*(=*γnq* ^2^/*C*_0_) of nonlinear capacitance at *α* = 1 (load-free) and $\overline{P}\mathrm{=1/2}$ to the linear capacitance *C*_0_ is assumed to be unity, i.e. *βnq* ^2^/4 = *C*_0_ (Notice $\gamma =\beta \overline{P}\mathrm{(1}-\overline{P})$ is maximized at $\overline{P}\mathrm{=1/2}$). Filled red circles indicate frequencies and the corresponding values of the membrane capacitance that maximize the power output. Other parameter values assumed are, *α* ^2^ = 1.2 and *ω*_*η*_/*ω*_*r*_ = 6, which is smaller than more realistic ratios (See Discussion). Traces respectively correspond to the values of $\overline{P}\mathrm{(1}-\overline{P})$: 0.25 (red), 0.13 (blue), and 0.06 (black), showing the dependence on the holding potential. The dotted line indicates the level of *C*_0_.

Eq. 6 indicates that nonlinear capacitance has its minimum at $\overline{\omega }=\sqrt{\alpha (\alpha +\mathrm{1/}{\overline{\omega }}_{\eta })}$. The minimum value of nonlinear capacitance is approximately $-\gamma n{q}^{2}{\overline{\omega }}_{\eta }\mathrm{/(2}\alpha )$ since $\mathrm{1/}{\overline{\omega }}_{\eta }\ll 1$. That leads to a condition

12 $$\zeta {\overline{\omega }}_{\eta }\, > \,2\alpha ,$$

under which negative nonlinear capacitance overwhelms the linear capacitance *C*_0_ and makes the membrane capacitance *C*_*m*_ negative. This condition practically determines the range of the membrane potential that satisfy *C*_*m*_ < 0. That is because the ratio *ζ*(=*γnq* ^2^/*C*_0_) includes a factor $\overline{P}\mathrm{(1}-\overline{P})$ in *γ* and because experimental data show that the peak nonlinear capacitance (at $\overline{P}=\mathrm{1/2}$) under load-free condition is as large as the linear capacitance for OHCs. In other words, as far as prestin motor is sensitive to voltage changes, i.e. $\overline{P}\mathrm{(1}-\overline{P})$ is not small, there is a frequency range where the membrane capacitance is negative (See Fig. 4).

In such cases, the frequency maximizing power output, ${\overline{\omega }}^{2}={\alpha }^{2}+\zeta -\mathrm{1/(2}{\overline{\omega }}_{\eta }^{2})$, is just outside the negative membrane capacitance region. Under this condition we have

13 $$\frac{{C}_{0}+{C}_{nl}}{{C}_{0}}\approx \frac{2{\alpha }^{2}+\zeta }{2{\zeta }^{2}}\cdot \frac{1}{{\overline{\omega }}_{\eta }^{2}},$$

which means the membrane potential is very small at the frequency of maximum power output (Fig. 4).

### Frequency Limit

The results obtained for our simple model system (Fig. 1) can be examined for implications to the mammalian cochlea by adding two assumptions[19]: that the output of OHC feeds back to hair bundle displacement and that the major source of the drag is the shear in the gap between the tectorial membrane and the reticular lamina, which is essential for hair bundle stimulation.

Hair bundle stimulation gives rise to changes $\hat{r}$ in normalized hair bundle resistance, which leads to cell displacement of the amplitude *x*(=*anp* ⋅ *k*/(*k* + *K*)), where *p* is described by Eq. 9. If the resulting cell displacements feed back to hair bundle stimulation, the cell functions as an amplifier that works against drag. Here we assume these changes are small and their final amplitudes, which depend on the nonlinearity of the system, are not considered.

Let us assume that hair bundle displacement *z* and OHC displacement *x* are proportional and described by *z* = *λx*. The dependence of the change $\hat{r}$ in hair bundle resistance on hair bundle displacement *z* has been experimentally studied. Let *g* be the sensitivity of the hair bundle transducer. Although the relationship between *z* and $\hat{r}$ is nonlinear, let *g* be the mechanosensitivity at the operating point. Then a condition for an effective amplifier is given by $g\lambda |x{|}_{(max)}\ge \hat{r},$ where |*x*| is expressed by using Eq. 9 for high frequencies,

14 $$|x|=\frac{\zeta a{i}_{0}}{{\omega }_{r}q}\frac{k}{k+K}\sqrt{H(\overline{\omega })}\cdot \hat{r},$$

with $H(\overline{\omega })=\mathrm{1/\{}{\overline{\omega }}^{2}[({\alpha }^{2}+\zeta -{\overline{\omega }}^{2}{)}^{2}+{(\lambda \overline{\omega }/{\overline{\omega }}_{\eta })}^{2}\mathrm{]\}}.$ Here *λ* appears in the denominator because it changes the amplitude of movement and in effect changes the drag coefficient. Unless $\mathrm{(2}-\sqrt{3})({\alpha }^{2}+\zeta )\, < \,\mathrm{1/}{\overline{\omega }}_{\eta }^{2}\, < \,\mathrm{(2}+\sqrt{3})({\alpha }^{2}+\zeta )$, the function $H(\overline{\omega })$ is a monotonically decreasing function of ${\overline{\omega }}^{2}$.

**Figure 5:**
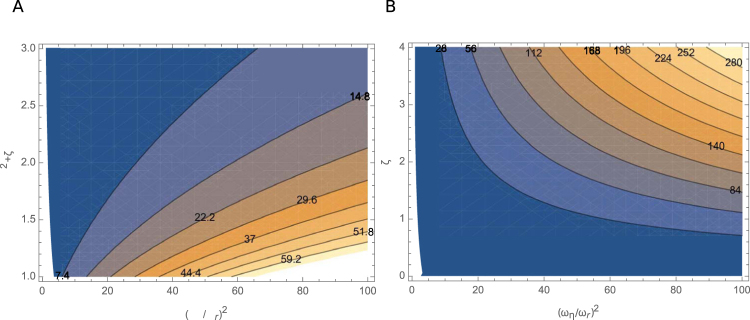
Contour plots of *H*_*max*_ and *ζ* ^2^ *H*_*max*_ for *λ* = 1. (**A**) Contour plot of *H*_*max*_ for *λ* = 1. Ordinate axis: ${\overline{\omega }}_{\eta }^{2}=({\overline{\omega }}_{\eta }/{\overline{\omega }}_{r}{)}^{2}$; abscissa: *α* ^2^ + *ζ*. The values of *H*_*max*_ are indicated in the plot. Brighter shades indicate higher values. (**B**) Contour plot of (*α* ^2^ + *ζ*)*ζ* ^2^ *H*_*max*_ assuming *α* ^2^ = 1.1, corresponding to a 10 kHz cell (see text). Ordinate axis: ${\overline{\omega }}_{\eta }^{2}$; abscissa: *ζ*. The values of (*α* ^2^ + *ζ*)*ζ* ^2^ *H*_*max*_ are indicated in the plot. Brighter shades indicate higher values.

If the transfer function *g*(*z*) is linearized to $\hat{r}=gz$ in the immediate neighborhood of the operating point, the frequency limit *ω*_*b*_ is expressed by

15 $${\omega }_{b}^{2} < {\zeta }^{2}({\alpha }^{2}+\zeta ){(\lambda g{i}_{0}\frac{a}{q}\frac{k}{k+K})}^{2}{H}_{max}({\alpha }^{2}+\zeta ,{\overline{\omega }}_{\eta }/\lambda ),$$

where the best frequency *ω*_*b*_ is related to the mechanical resonance frequency *ω*_*r*_ by ${\omega }_{b}^{2}=({\alpha }^{2}+\zeta ){\omega }_{r}^{2}$. The local maximum of $H(\overline{\omega })$ is expressed by ${H}_{max}({\alpha }^{2}+\zeta ,{\overline{\omega }}_{\eta }/\lambda )$. The dependence of this function on the two parameters is plotted as a contour graph (Fig. 5).

This inequality indicates the importance of the ratio *k*/(*k* + *K*). While a larger value of *K* elevates the mechanical resonance frequency *ω*_*r*_, it reduces *ω*_*b*_, making the effectiveness of higher frequency unfavorable. This issue will be discussed later.

### Values of the Parameters for a 10 kHz cell

Reliable parameter values are available for 10 kHz cells, if we can assume that the properties of OHCs in the this frequency region of the cochlea in guinea pigs are similar to those in rats and gerbils.

First, examine the condition ${\omega }_{\eta }/{\omega }_{r}\gg 1$, which was used throughout the derivation and also for optimizing $H(\overline{\omega })$. Assume that the source of the major drag is shear in the gap between the reticular lamina and the tectorial membrane. Then the drag coefficient *η* is given by *η* = *μS* ^2^/*d*, where *μ* is the viscosity of the fluid, *S* the surface area, and *d* the gap. If *S* = 10 *μ*m × 20 *μ*m, and *d* = 1 *μ*m[19], *η* = 1.6 × 10^−7^ N/m, using the viscosity of water.

Given the axial elastic modulus of 510 nm/unit per strain^32^, a 20 *μ*m long OHC has stiffness *k* of 2.6 × 10^−2^ N/m. Even without an external elastic load, we obtain *ω*_*η*_ ≈ 1.5 × 10^6^, much higher than the auditory frequency. Thus the condition ${\omega }_{\eta }/{\omega }_{r}\gg 1$ holds. For shorter cells of higher frequency region the cell stiffness *k* is higher, inversely proportional to cell length. The gap drag *η* is also higher, inversely proportional to the hair bundle length.

Now let us examine the frequency limit. For a 20 *μ*m long cell, typical of the 10 kHz cochlear region, the linear capacitance is *C*_0_ = 8 pF and *an* = 1 *μ*m, which is 5% of the cell length. Most *in vitro* experiments show the unitary motile charge of *q* = 0.8 *e*, where *e* is the electronic charge. The membrane potential ($\overline{V}$) is near the optimal range ($\overline{P}\approx \mathrm{1/2}$) for the motile element. The resting basolateral resistance is 7 MΩ and the resting membrane potential of −50 mV requires the resting apical resistance of 30 MΩ^22^. These values lead to *i*_0_ = 4 nA.

The sensitivity *g* of hair bundles determined by *in vitro* experiments tend to be underestimates due to the matching of the force probe with hair bundles^33^. For this reason, *g* = 1/(25 nm)^34^ is taken.

If we can assume *k*/(*k* + *K*) = 0.1 together with *λ* = 1 and *H*_*max*_ = 20, an underestimate (see Fig. 5A), we obtain *f*_*b*_ = *ω*_*b*_/2*π* < 1.1 × 10^4^, consistent with the location of 10 kHz. Power output can be evaluated using this set of parameters. With this set of parameter values, a typical value for maximal power output would be 0.1 fW for $\hat{r}\mathrm{=0.1}$. An extrapolation to the maximal output is 10 fW. These values are in a reasonable agreement with the expected output range of a single 10 kHz cell estimated from cochlear mechanics^35,36^.

## Discussion

For an OHC to be effective at higher frequencies, two conditions should be met. One is that the mechanical resonance frequency ${\omega }_{r}(=\sqrt{(k+K)/m})$ must be compatible with those frequencies. The other is *ω*_*b*_, which is proportional to *k*/(*k* + *K*), must be larger than *ω*_*r*_. For this reason if *k*/(*k* + *K*) = 0.1 for a 10 kHz cell, an OHC cannot be effective at 100 kHz, as shown in the following.

The membrane resistance decreases about 3-fold for a 10-fold increase in the frequency of cell location^22^, contributing to a 3-fold increase in the limiting frequency. A 10-fold increase of *ω*_*r*_ requires a 100-fold increase in the ratio (*k* + *K*)/*m*. Since each OHC is held by Deiters’ cup in at the base around the nucleus, the difference in the stiffness *k* between a 5 *μ*m cell and a 20 *μ*m cell is about 10 fold, much less than a 100-fold difference in basilar membrane stiffness^37,38^. A 10-fold increase in the frequency reduces the thickness of boundary layer by $1/\sqrt{10}$-fold. This factor may lead to factor up to $\sim 3$ in reducing the mass *m*, to which the boundary layer of the fluid contributes. Thus, the ratio *k*/*m* increases $\sim 30$-fold at most, leading to a value 0.03 for *k*/(*k* + *K*), which barely supports 10 kHz resonance.

If resonance at 10 kHz is achieved without the external elastic load, a condition *K* > 2.3*k* to achieve a 100-fold increase in (*k* + *K*)/*m*. This leads to 0.3 for the stiffness ratio *k*/(*k* + *K*), allowing a limiting frequency above 100 KHz, despite a decrease in *H*_*max*_ due to a 10-fold increase in *k*.

Another factor is the ratio *ζ*(=*C*_*nl*_/*C*_0_) (Fig. 5B). A two-fold increase in *ζ* may lead to an additional 70% increase in the limiting frequency. Guinea pig data indeed shows a 4-fold increase in *ζ* (at the capacitance peak) from low frequency cells (*C*_0_ = 35 pF) to high frequency cells (5 pF)^39^. However, rat data contradict this observation^40^.

Other factors include the amplitude ratio *λ*, hair bundle sensitivity *g*, and the molecular characteristic *a*/*q* of the motile element. If those factors do not significantly differ at higher frequency locations, the ratio *k*/(*k* + *K*) must remain relatively large. Since OHCs should be involved in a relative motion between the basilar membrane (BM) and the reticular lamina (RL)^41,42^, the effectiveness of OHC requires that the resonance frequency of this relative motion must be close to that of the local BM. Since the cell bodies of OHCs would be much less stiff than the BM, the associated mass must be much smaller. Then, transfer of energy between the modes is likely.

An argument against multiple modes of motion could be made by assuming that the origin of the elastic load is the BM. Indeed, the analysis of resonance at ~10 kHz may give such an impression. Cochlear mechanics then suggests that the main origin of the inertia is fluid mass and that the ratio of the stiffness and the mass is not an issue. That is because an examination of energy flow indicates that the impedances due to the stiffness of the BM and fluid mass are equal and opposite, canceling each other at all frequencies and locations where the traveling wave is present^43^. Single mode of motion, therefore, would suffice. However, the starting assumption of such a counterargument can be questioned because there is no clear justification that the BM is the source of the elastic load on OHCs, which connect the BM and the RL,and the RL appears more compliant than the BM.

In the advent of technological innovation, which allows us to observe the displacement of each component in the cochlear partition^41^, the issue of modes of motion in the cochlea is of great interest to understand the detailed mechanism of the cochlear amplifier^44^, in which OHCs play a key role.

Finally, the existence of predicted negative capacitance could be tested by *in vitro* experiments. Inertial and elastic loads can be imposed on an OHC by engaging an elastic probe to the cell. For example, a mechanical resonance at frequency of ~100 Hz has been observed in such a system (Fig. 2 of ref.^11^).
